# KIAA1429 mediates epithelial mesenchymal transition in sorafenib‐resistant hepatocellular carcinoma through m6A methylation modification

**DOI:** 10.1002/cam4.5432

**Published:** 2022-11-24

**Authors:** Ye Kuang, Yun Cheng, Jia Wang, Hongyan Li, Xianghong Cao, Yang Wang

**Affiliations:** ^1^ Yan'An Hospital Medical Laboratory Kunming China

**Keywords:** EMT, hepatocellular carcinoma, KIAA1429, m6A methylation, sorafenib resistance

## Abstract

**Background:**

Hepatocellular carcinoma (HCC) is a primary liver cancer with high mortality. The long‐term use of sorafenib, a targeted drug for hepatocellular carcinoma, will lead to drug resistance, and patients are prone to cancer metastasis, the molecular mechanism of which is still unclear.

**Methods:**

In our study, we constructed a sorafenib‐resistant hepatocellular carcinoma cell line (HepG2/Sora) and validated the resistance in vivo and in vitro. Transwell assays confirmed the invasion and migration abilities of cells. Colorimetric assays confirmed that the level of m6A methylation modification in cells. RT‐qPCR and Western blot assays confirmed that the expression levels of KIAA1429 in HepG2/Sora cells and tissues. The EMT related proteins were detected by Western blot assay.

**Results:**

Transwell and Western blot assays confirmed that HepG2/Sora cells had higher invasion and migration abilities and showed EMT phenomena. Colorimetric assays confirmed that the level of m6A methylation modification was upregulated in HepG2/Sora cells. Transwell and Western blot assays confirmed that inhibiting m6A methylation in HepG2/Sora cells inhibited their invasion, migration ability and EMT phenomenon. RT‐qPCR and Western blot assays confirmed that the expression levels of KIAA1429 in HepG2/Sora cells and tissues was increased. Silencing KIAA1429 in HepG2/Sora cells inhibited their invasion, migration ability and EMT phenomenon. Finally, we found that the medium supernatant of sorafenib‐resistant HepG2/Sora cells induced vascular production of EA.hy926 cells, and silencing KIAA1429 inhibited this induction effect.

**Conclusion:**

We suggest that KIAA1429 promotes sorafenib‐resistant hepatocellular carcinoma invasion, migration and EMT by mediating m6A methylation. KIAA1429 with its mediated m6A methylation may be a key factor for sorafenib‐resistant patients prone to cancer cell metastasis.

## Graphical abstract

KIAA1429 mediated m6A methylation promotes the invasion, migration and EMT of Sorafenib resistant liver cancer cells

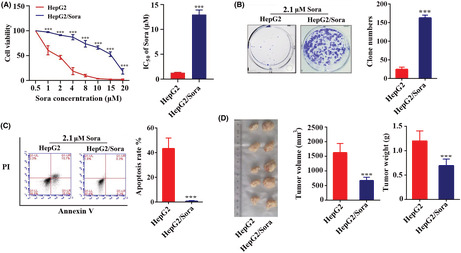

## 1 INSTRUCTION

Hepatocellular carcinoma (HCC) is a primary liver cancer with high mortality.^1^ It is one of the most common malignant tumors in the world and the third most common cause of cancer‐related death.^2^ Sorafenib is a new drug for molecular targeted therapy that can play simultaneous, the dual roles in anti angiogenesis and antitumor cell proliferation. Sorafenib has resulted in breakthrough in the clinical research of the treatment on liver cancer, as it effectively prevents the deterioration of the disease and significantly prolongs the survival time of patients with advanced liver cancer.^3^, ^4^ Therefore, the EU drug administration (October 2007) and the US Food and Drug Administration (November 2007) approved sorafenib for the treatment of unresectable hepatocellular carcinoma.^5^

Although the average overall survival of patients treated with sorafenib is significantly improved, hepatocellular carcinoma is prone to metastasis and EMT after sorafenib resistance develops, which limits the benefit of sorafenib therapy.^6^ Recent studies have found that tumor resistance is closely related to epithelial mesenchymal transformation (EMT) of tumor cells. Increasing attention has been given to the role of EMT in drug resistance of antitumor therapy. A large number of studies have shown that after the cells undergo EMT transformation, they will assume an anti‐apoptotic effect, which will lead to drug tolerance, and long‐term treatment with chemotherapy drugs will also induce the EMT in tumor cells.^7^, ^8^ Shunjie Xia et al.^9^ reported that sorafenib‐resistant HCC cells showed significant mesenchymal phenotype and stem cell characteristics.

EMT is regarded as a pathological process leading to tumor progression. In the process of malignant evolution of tumors, EMT induces invasion, metastasis, and immune escape in tumor cells.^10^ Therefore, the occurrence of EMT predicts the malignant process of tumors.^11^, ^12^ N6 methyladenosine (m6A) is a base modification behavior that widely exists in mRNA, and is used to maintain its stability of mRNA.^13^, ^14^ Currently, an increasing number of studies show that tumor cells are regulated by RNA m6A modification in the process of EMT. For example, Lin X et al.^15^ reported that m6A of mRNA was significantly upregulated during the EMT in tumor cells, and was able to promote the invasion and metastasis by increasing the translation of Snail. Therefore, we suggest that m6A methylation is involved in the regulation of EMT in sorafenib‐resistant hepatocellular carcinoma, and we believe that EMT caused by drug resistance is the key factor for patients with sorafenib resistance in the development of cancer metastasis.

In our study, we first constructed the sorafenib‐resistant human hepatocellular carcinoma cells (HepG2/Sora) and verified their drug resistance. We found that HepG2/Sora cells showed a high degree of invasion and migration, accompanied by EMT phenomenon. We found that m6A methylation level was upregulated in sorafenib‐resistant HepG2/Sora cells and was associated with cell invasion, migration and EMT. We further found that among m6A methylation and demethylase, only the mRNA and protein expression levels of KIAA1429 were upregulated in HepG2/Sora cells. Silencing KIAA1429 inhibited the invasion and migration of HepG2/Sora cells. Finally, we found that the medium supernatant of sorafenib‐resistant HepG2/Sora cells induced vascular production of human umbilical vein endothelial cell fusion cells (EA.hy926) and silencing KIAA1429 inhibited this induction effect. In conclusion, we suggest that KIAA1429 mediated m6A methylation promotes the invasion, migration and EMT of sorafenib‐resistant HCC. Therefore, the significance of our study is to reveal the molecular mechanism of KIAA1429 mediated EMT in sorafenib‐resistant HCC through m6A methylation modification. At the same time, KIAA1429 and its m6A methylation may be the key factors for sorafenib‐resistant patients to be prone to tumor metastasis.

## 2 MATERIALS AND METHODS

### 2.1 Cell lines

A human hepatocellular carcinoma cells line (HepG2; cat. no. TCHu 72) and human umbilical vein endothelial cell fusion cells (EA.hy926; cat. no. GNHu39) were purchased from the National Collection of Authenticated Cell Cultures. HepG2 cells and EA.hy926 cells were cultured in Dulbecco's modified Eagle medium (DMEM; cat. no.11965092; Thermo Fisher Scientific, Inc.) or Roswell Park Memorial Institute 1640 medium (cat. no.11875085; Thermo Fisher Scientific, Inc.) containing 10% fetal bovine serum (cat. no. 10099141; Thermo Fisher Scientific, Inc.) and 1% penicillin–streptomycin solution (cat.no. C0222; Beyotime Inc.). The cell culture conditions were 37°C and 5% CO_2_.

### 2.2 Sorafenib‐resistant HepG2 cell conduction

HepG2 cells in logarithmic growth phase were treated with 0.5, 1 or 1.5 μg/mL sorafenib (cat.no. Y0002098; Sigma‐Aldrich LLC.). After 48 h of drug stimulation, the medium was replaced DMEM with 10% fetal bovine serum. After the cells returned to normal morphology, drugs were added for induction. The above steps were repeated until the cells were adapted to this concentration and grew normally. The cells were stimulated with increasing dosage of sorafenib for 2 months. Finally, the sorafenib‐resistant HepG2 cells (HepG2/Sora) were able to grow normally in the presence of 2.1 μg/ml sorafenib.

### 2.3 KIAA1429 silencing in sorafenib‐resistant HepG2 cells

Endoribo nuclease prepared small interfering RNAs (esiRNAs) targeting human KIAA1429 (cat.no. EHU055891, Sigma‐Aldrich LLC.) were used to silence KIAA1429 in sorafenib‐resistant HepG2 cells (HepG2/Sora). EsiRNA targeting EGFP (cat.no. EHUEGFP; Sigma‐Aldrich LLC.) was used a negative control. The above constructs were transfected into HepG2/Sora cells using Lipofectamine™ LTX Reagent with PLUS™ Reagent (cat. no. A12621; Thermo Fisher Scientific, Inc.). siRNA(20 pmol) was added to 50 μl Opti‐MEM (cat. no. 11058021; Thermo Fisher Scientific, Inc.) medium without serum. Lipofectamine (1 μl, cat. no. A12621; Thermo Fisher Scientific, Inc.) was also added to 50 μl Opti‐MEM serum‐free medium. The tubes were mixed and placed at room temperature for 20 min to form the complexes. Each mixture was added to a HepG2/Sora cell suspension, which was cultured at 37°C, 5% CO_2_. After 48 h, other experimental steps were carried out. HepG2/Sora cells were stably transfected with siRNA negative control (siNC) or siRNA KIAA1429 (siKIAA1429). siRNA KIAA1429: GTGACCTTGCCTCACCAACTGCACTTCTGATTATGAGAAC TGTGTTGGATTTGATTGTAGAAGACTTGCAAAGCACTTCAGAAGATAAAGAAAAACAGTATACTAGCCAAACCACCAGGTTGCTTGCTCTTCTTGATGCTCTGGCTTCACACAAAGCTTGTAAATTAGCTATTTTGCATCTAATTAATGGAACTATTAAAGGTGATGAAAGATATGCAGAGATATTCCAGGATCTTTTAGCTTTGGTGCGGTCTCCTGGAGACAGTGTTATTCGCCAACAGTGTGTTGAATATGTCACATCCATTTTGCAGTCTCTCTGTGATCAGGACATTGCACTTATCTTACCAAGCTCTTCTGAAGGTTCTATTTCTGAACTGGAGCAGCTCTCCAATTCTCTACCAAATAAAGAATTGATGACCTCAATCTGTGACTGTCTGTTGGCTACGC.

### 2.4 Tumor xenograft model

Six‐week‐old BALB/c‐nu mice (*n* = 10) were purchased from Hunan STA Laboratory Animal Co., Ltd. Nude mice were adaptively fed in a specific pathogen free (SPF) environment for 7 days. The study protocol was ethically approved by the Kunming Yan'an Hospital Experimental Animal Ethics Committee (Kunming, China; approval no. 2020004). Mice were randomly divided into a control group (HepG2) and an experimental group (HepG2/Sora) with 5 mice in each group. A cell suspension (4 × 10^6^ cells per mouse) was injected into the right lateral thighs of mice after light anesthesia using 37.5 mg/kg pelltobarbitalum natricum (cat.no. P‐010; Sigma‐Aldrich LLC.). The drug treatment was carried out when the tumor size was approximately 100 mm^3^. Sorafenib was prepared with 0.4% DMSO+PBS solution and administered to mice by intraperitoneal injection at a dose of 100 mg/kg after light anesthesia using 37.5 mg/kg pelltobarbitalum natricum. Sorafenib was administered once a day for 5 consecutive days. The physical state of the nude mice was observed and recorded every day. Mice in poor condition were terminated in time and euthanized immediately. All the mice were sacrificed using intraperitoneal injection of 200 mg/kg pelltobarbitalum natricum 5 days after sorafenib administration. Before euthanasia, the mice were given oral administration of ibuprofen (40 mg/kg; cat.no.14883; Sigma‐Aldrich LLC.) with water to relieve pain. The tumors were removed surgically and photographed using a camera (Model: a7s3; Sony Corporation).

### 2.5 Real‐time quantitative PCR (RT‐qPCR)

RNAs of cells and tissues was extracted using the Total RNA Extraction Kit (cat.no.R1200; Beijing Solarbio Science & Technology Co., Ltd.). The overall experimental operation was carried out accordance with the product instructions. The RNA of each group was reverse transcribed in equal amounts (2 μg) using a Universal RT‐PCR Kit(M‐MLV) (cat.no.RP1100;Beijing Solarbio Science & Technology Co., Ltd.). The reverse transcription conditions were as follows: 42°C for 1 min; 80°C for 5 min; and 4°C for 5 min. qPCR assays were performed using a SsoAdvanced Universal SYBR‐Green Supermix (cat.no.172–5270; Bio‐Rad Laboratories, Inc.) in accordance with the product instructions. The qPCR conditions were as follows: predenaturation (95°C for 30 s, 1 cycle); denaturation and annealing (95°C for 15 s, 60°C for 30 s, 40 cycles); dissociation curve (65°C–95°C; 0.5°C/2 s). The following primer sequences were used: KIAA1429 forward, 5’‐GGGATGGGACAGTAGCAACAA‐3′ and reverse, 5’‐TAATGTGGGGTGAAGGAGCAG‐3′; Methyltransferase 3 (METTL3) forward, 5’‐AAAATGTGGAAGCTTTGGAGGC‐3′ and reverse, 5’‐AGGAACACTGCTTGGTGAGC‐3′; Methyltransferase 14 (METTL14) forward, 5’‐GCACAGACGGGGACTTCATT‐3′ and reverse, 5’‐ACACAGCACCATGTCCTATTTC‐3′; WT1 associated protein (WTAP) forward, 5’‐ATGGCGAAGTGTCGAATGCT‐3′ and reverse, 5’‐CAAACCCCTTACCATCCTGACT‐3′; RNA binding motif protein 15 (RBM15) forward, 5’‐GAGAAAACTTGGCGCTGACC‐3′ and reverse, 5’‐AAACAGCCAAAGAACACTTCAG‐3′; Alpha‐ketoglutarate dependent dioxygenase (FTO) forward, 5’‐AGAACTACATGCAGGAGGCG‐3′ and reverse, 5’‐GGAGCCCGACATACCTTAGC‐3′; AlkB homolog 5, RNA demethylase (ALKBH5) forward, 5’‐ATTAGATGCACCCCGGTTGG‐3′ and reverse, 5’‐AGCAAGCCAAGGCTCCTAAA‐3′; glyceraldehyde‐3‐phosphate dehydrogenase (GAPDH) forward, 5’‐GGAGCGAGATCCCTCCAAAAT‐3′ and reverse, 5’‐GGCTGTTGTCATACTTCTCATGG‐3′. GAPDH was the internal reference for the RT‐qPCR assay. The relative mRNA expression levels were calculated using the 2^−ΔΔCq^ method.^16^

### 2.6 Western blot

Protein lysates were treated with protease inhibitors and phosphatase inhibitors (cat.no. C2501 and S0143; HaiGene Biotech Co., Ltd.), and incubated on ice for 30 min for protein extraction from the tissues and cells. A BCA Protein Assay Kit (cat.no. PC0020; Beijing Solarbio Science & Technology Co., Ltd.) was used to detect the concentration of the extracted protein. SDS‐PAGE was used to separate equal amounts of proteins (5 μg) with different molecular weights. The proteins were transferred to PVDF membranes from SDS‐PAGE gels and blocked with BSA buffer (cat.no. P0252; Beyotime Inc.) at room temperature for 2 h. The PVDF membrane was incubated with primary antibody at 4°C overnight. The membranes were washed 3 times with Tris‐buffered saline and Tween 20 (TBST) for 10 min each time. The PVDF membrane was incubated with secondary antibody at room temperature for 2 h. The membrane was incubated with ECL solution in the dark for 10 min and then imaged in a gel imaging system (Model: ChemiDoc XRS+; Bio‐Rad Laboratories, Inc.). Relative quantification based on the gray value of the strips was performed used Adobe Photoshop (Adobe Systems Software Ireland Ltd.). Antibodies against Slug (ab27568), E‐cadherin (ab152102), Snail (ab82846), KIAA1429 (ab271136) and β‐actin (ab8227) were purchased from Abcam.

### 2.7 Cell counting kit‐8 (CCK‐8) assay

Cells were seeded in 96‐well plates at 5000 cells/100 μl. Ten microliters of CCK‐8 solution was added to a 96‐well cell culture plate and incubated in a 37°C incubator for 1 h. The absorbance value of each well was detected at wavelength of 450 nm using a microplate photometer (Multiskan FC; Thermo Fisher Scientific, Inc.). The cell survival rate was calculated as follows: [(As‐Ab) / (Ac‐Ab)] × 100% (As: absorbance of experimental hole; Ac: absorbance of control hole; Ab: absorbance of blank hole). The half maximal inhibitory concentration (IC_50_) was calculated by Statistical Product Service Solutions (SPSS) software (version:22.0; International Business Machines Corporation). Resistance coefficient (RI) = Resistant strain IC_50_/Sensitive strain IC_50_.

### 2.8 Colony forming assay

HepG2 and HepG2/sora cells in the logarithmic growth stage were prepared into cell suspensions and inoculated into 6‐well culture plates (300 cells per well). After the cells adhered to the wells, the medium was replaced with DMEM supplemented with 1.28 μM sorafenib and changed every 3 days. After 14 days of continuous culture, the clonal culture was stopped. The medium was removed and methanol was added to fix the cells at room temperature for 15 min. The fixed cell clones were stained with 0.1% crystal violet (cat.no. Y0000418; Sigma‐Aldrich LLC.) at room temperature for 10 min. The results of clone formation were photographed and counted.

### 2.9 Transwell assay

The migration and invasion capacities of HepG2 and HepG2/sora cells were assessed using Transwell chambers (cat.no. RTN70; Sigma‐Aldrich LLC.) in 24‐well plates. HepG2 and HepG2/Sora cells in serum‐free DMEM medium were cultured in the upper chamber of a Transwell with or without Matrigel, while the lower chamber was filled with DMEM medium containing 10% FBS. After 24 h of cell culture at 37°C, the cells in the upper portion of the chamber were removed and the cells in the lower portion of the chamber were remained. The migrating and invading cells were fixed with 20% methanol and stained with 0.1% crystal violet. The staining results were photographed under a microscope at 200× magnification in five random fields. Crystal violet was dissolved in acetic acid, and the absorbance value was detected at OD_570_ for relative quantitative analysis.

### 2.10 Tubule formation experiment

A precooled 96‐well plate was placed on ice, and 50 μl Matrigel (cat.no. A1569601; Thermo Fisher Scientific, Inc.) was added to each well, which was solidified at 37°C for 1 h. EA.hy926 cells were serum‐starved for 24 h. The cells were resuspended to 10^5^ cells/ml in HepG2/Sora‐siNC or HepG2/Sora‐siKIAA1429 culture supernatant. A total of 100 μl EA.hy926 cell suspension was added to a 96‐well culture plate containing Matricel. After approximately 4 h of culture, the tubular structure appeared, and 5–6 visual fields were randomly selected to take photos. Image J (verson 1.48; National Institutes of Health) software was used to determine the tubule length.

### 2.11 Cell apoptosis assay

HepG2 cells and HepG2/Sora cells treated with 1.28 μM were centrifuged to remove the culture medium, and the cells were collected and repeatedly washed with PBS. The cells were resuspended in 500 μl Annexin V‐PI binding solution (cat.no. C1062S; Beyotime Inc.). Cell suspension added with 10 μl PI and 5 μl Annexin V‐FITC was gently mixed and incubated at room temperature for 10 min in the dark. Channel 1 (Annexin V) and channel 2 (PI) were selected for detection by flow cytometry (model: Accuri C6; Becton, Dickinson and Company).

### 2.12 m6A RNA methylation quantification assay

m6A RNA methylation quantification of HepG2 cells and HepG2/Sora cells was performed using the EpiQuik m6A RNA Methylation Quantification Kit (cat.no. P‐9005; EpiGentek Group Inc.), according to the manufacturer's instructions. Relative m6A RNA methylation was expressed as the percentage of m6A in total RNA.

### 2.13 Statistical analysis

SPSS 15.0 software (International Business Machines Corporation, Inc.) was used for the statistical analysis. All experiments in the study were repeated at least 3 times and the data were presented as the mean ± SD (standard deviation). The experimental results were statistically analyzed by a two‐tailed unpaired Student's *t*‐test. One‐way ANOVA with the least significant difference post hoc test was used for the comparison of multiple groups. *p* < 0.05 was defined as statistically significant experimental results.

## 3 RESULTS

### 3.1 Verification of drug resistance in a sorafenib resistant hepatocellular carcinoma cell line

**FIGURE 1:**
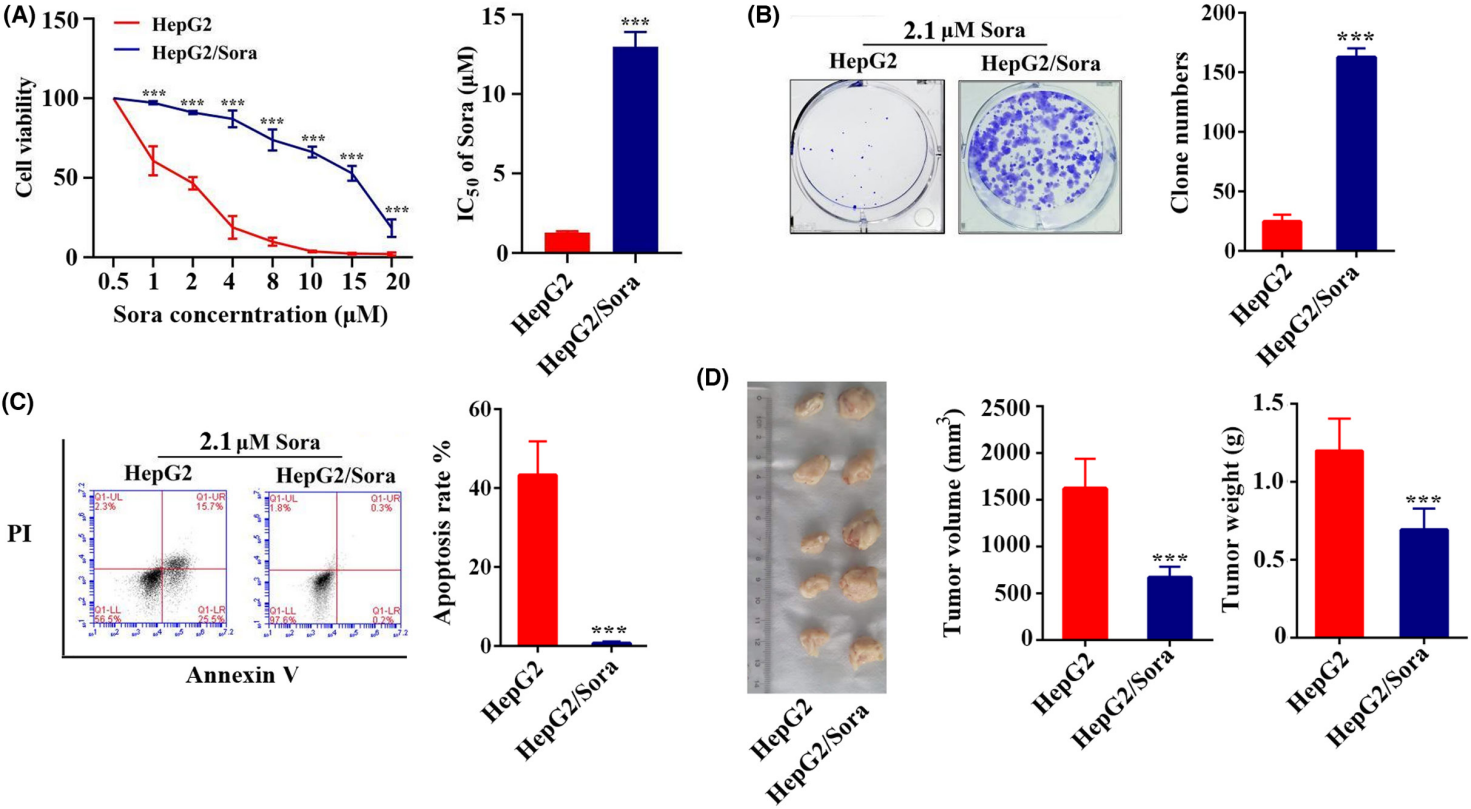
Verification of the drug resistance of sorafenib‐resistant strains. (A) CCK‐8 assay (left). Cell viability in HepG2 or HepG2/Sora cells treated with different concerntration of sorafenib. HepG2 groups vs. HepG2/Sora groups, ***p < 0.001. (B) The IC_50_ values of sorafenib in HepG2 and HepG2/Sora cells to were calculated by SPSS. HepG2 groups vs. HepG2/Sora groups, ***p < 0.001. (B) HepG2 and HepG2/Sora cells were treated with 2.1 μM sorafenib for the clone formation assay. The results of the experiment were photographed (no magnification) and counted. HepG2 groups vs. HepG2/Sora groups, ***p < 0.001. (C) Annexin V‐PI assay. Scatter plot of flow cytometry (left) and apoptosis rate in HepG2 or HepG2/Sora cells treated with 2.1 μM sorafenib. HepG2 groups vs. HepG2/Sora groups, ***p < 0.001. (D) The xenograft model was used to identify the sorafenib resistance of HepG2/Sora cells (left). Tumor volume and tumor weight in the HepG2 groups or HepG2/Sora groups (middle and right). HepG2 groups vs. HepG2/Sora groups, ***p < 0.001.

The drug concentration and cell viability curves of HepG2 and HepG2/Sora cells were plotted using the CCK‐8 method, and IC_50_ values were calculated at 24 h. The IC_50_ values of HepG2 and HepG2/Sora cells were 1.28 ± 0.09 μM and 12.99 ± 0.75 μM respectively (Figure 1A). The drug resistance index of HepG2/Sora cells was 6.44 ± 1.3. As shown in Figure 1B, it was difficult for HepG2 cells to form clones under the stimulation of 2.1 μM sorafenib, while the clones in HepG2/Sora formed well. Cell apoptosis experiments showed that a large number amount of apoptosis occurred in HepG2 cells under 2.1 μM sorafenib treatment, accounting for 45.41 ± 6.48% of the total cells, while the proportion of apoptosis in HepG2/Sora cells was decreased, accounting for 1.12 ± 0.29% of the total cells (Figure 1C). The xenograft experiments in nude mice showed that the volume and weight of xenografts formed by HepG2/Sora cells were significantly higher than those formed by normal HepG2 cells treated with 2.1 μM sorafenib (Figure 1D).

### 3.2 The invasion and migration of HepG2 cells were enhanced after sorafenib resistance

**FIGURE 2:**
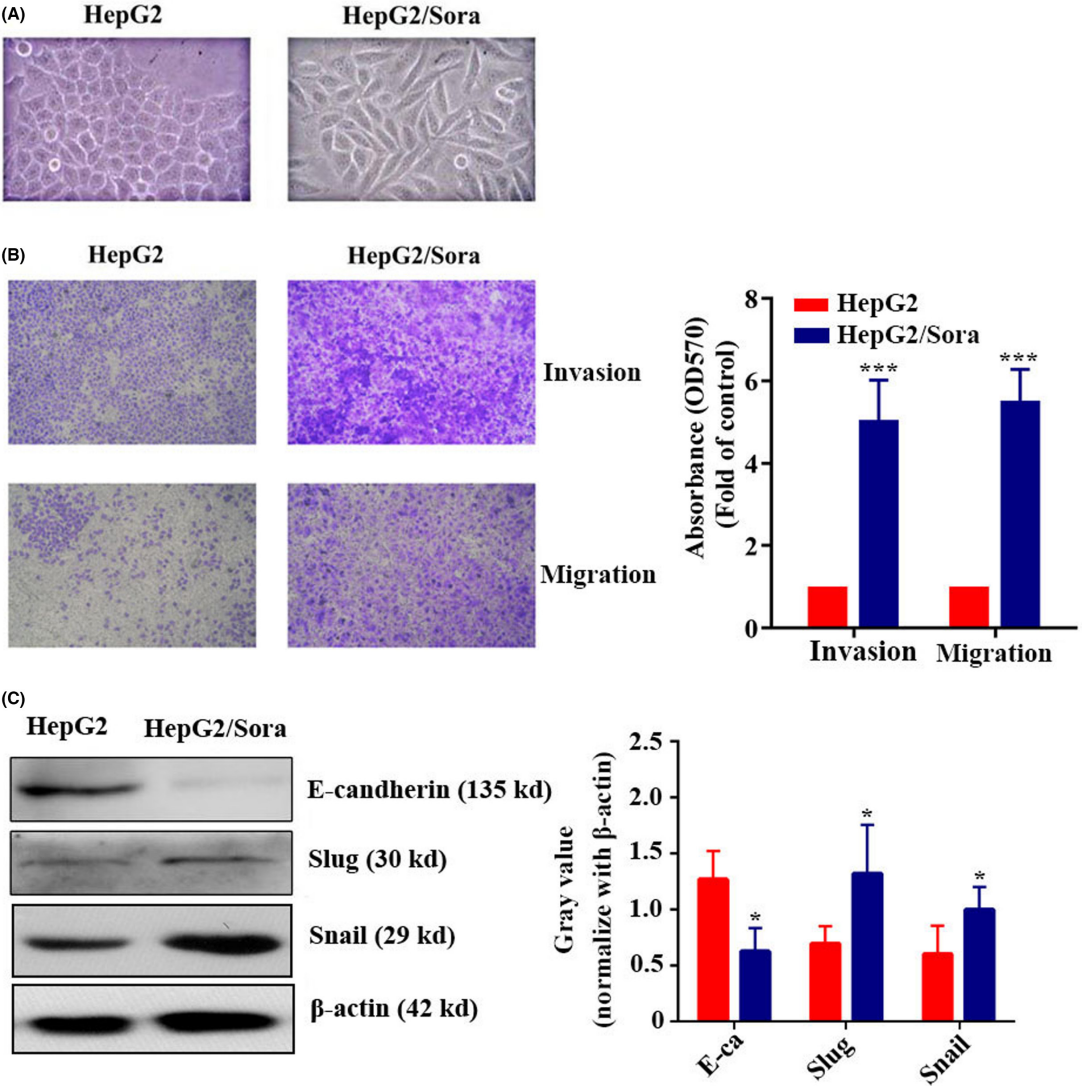
The invasion and migration of HepG2 cells were enhanced after sorafenib resistance. (A) Morphology of HepG2 or HepG2/Sora cells under the microscope (200× magnification). Transwell migration assay. Representative images (left) and quantification (right) of HepG2 or HepG2/Sora cell migration. HepG2 groups vs. HepG2/Sora groups, ***p < 0.001. (C) Western blot analysis of E‐cadherin, Slug, Snail and β‐Actin expression levels in HepG2 and HepG2/Sora cells (left). Relative gray values of E‐cadherin, Slug and Snail according to western blotting (right). HepG2 groups vs. HepG2/Sora groups, *p < 0.05.

We found that the HepG2/Sora cells showed EMT in terms of cell morphology. Compared with HepG2 cells, the HepG2/Sora cells transformed to a spindle shape, and became loose (Figure 2A). Transwell assays showed that HepG2/Sora cells showed higher migration and invasion abilities than HepG2 cells (Figure 2B). The crystal violet on the cells passing through the Transewell chamber was dissolved in acetic acid. As shown in Figure 2B, the absorbance of the crystal violet‐acetic acid solution from HepG2/Sora cells was significantly higher than that from HepG2 cells. The expression levels of EMT marker proteins (i.e., Slug, E‐cadherin and Snail) were detected by western blotting. As shown in Figure 2C, the protein expression of E‐cadherin protein was downregulated and the protein expression levels of Slug and Snail protein were upregulated in HepG2/Sora cells.

### 3.3 m6A methylation mediated invasion, migration and EMT of sorafenib‐resistant strains

**FIGURE 3:**
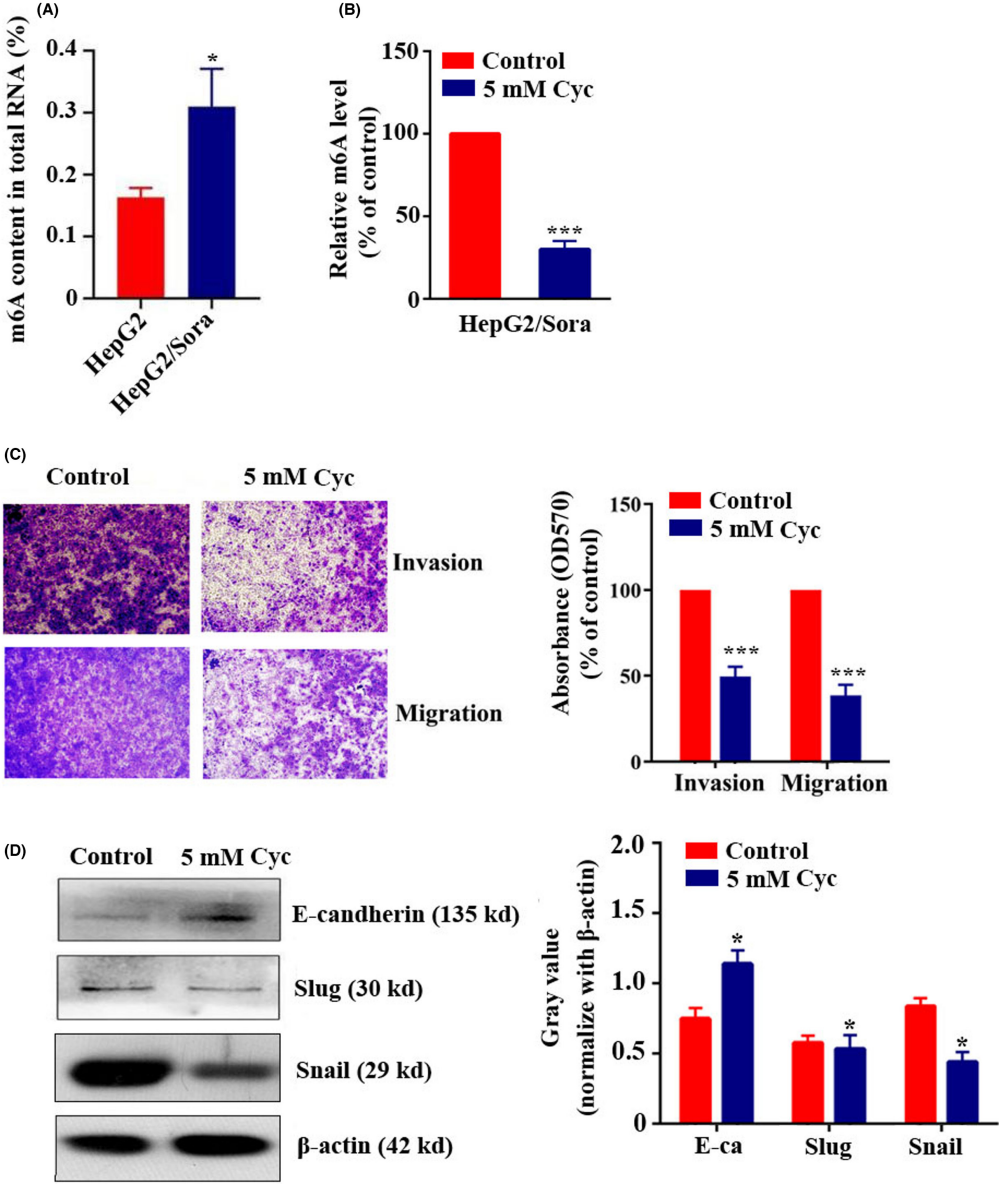
m6A Methylation mediated the invasion, migration and EMT of sorafenib‐resistant strains. (A) m6A content in total RNA in HepG2 and HepG2/Sora cells. HepG2 groups vs. HepG2/Sora groups, *p < 0.05. (B) m6A content in total RNA in HepG2/Sora cells with or without 5 mM Cyc treatment. Control groups vs. Cyc groups, ***p < 0.001. (C) Transwell assay. Representative images (left, 100× magnification) and quantification (right) of HepG2/Sora cell migration with or without 5 mM Cyc treatment. Control groups vs. Cyc groups, ***p < 0.001. (D) Western blot analysis of E‐cadherin, Slug, Snail and β‐Actin expression in HepG2/Sora cells with or without 5 mM Cyc treatment (left). Relative gray values of E‐cadherin, Slug and Snail according to western blotting (right). HepG2 groups vs. HepG2/Sora groups, *p < 0.05.

The m6A methylation levels of HepG2 and HepG2/Sora cells were detected by colorimetry. The results showed that the m6A methylation level of HepG2/Sora cells was significantly higher than that of HepG2 cells, which was 1.76 ± 0.35 (*p* < 0.05) times higher than that of HepG2 cells (Figure 3A). The m6A methylation level of HepG2/Sora cells was significantly inhibited by cycloleucine (cyc; Figure 3B). The results of the Transwell assay showed that the cell migration and invasion abilities of HepG2/Sora cells were decreased after treatment with 5 mM cycloleucine (Figure 3C). The crystal violet on the cells passing through the Transewell chamber was dissolved in acetic acid. As shown in Figure 3C, the absorbance of crystal violet‐acetic acid solution from HepG2/Sora cells treated with 5 mM cycloleucine was significantly lower than that from HepG2/Sora cells. Western blot results showed that HepG2/Sora cells treated with 5 mM cyc, the expression of E‐cadherin protein was upregulated, while the expression levels of Slug and Snail proteins were downregulated (Figure 3D).

### 3.4 KIAA1429 mediated the invasion, migration and EMT of Sorafenib resistant strains

**FIGURE 4:**
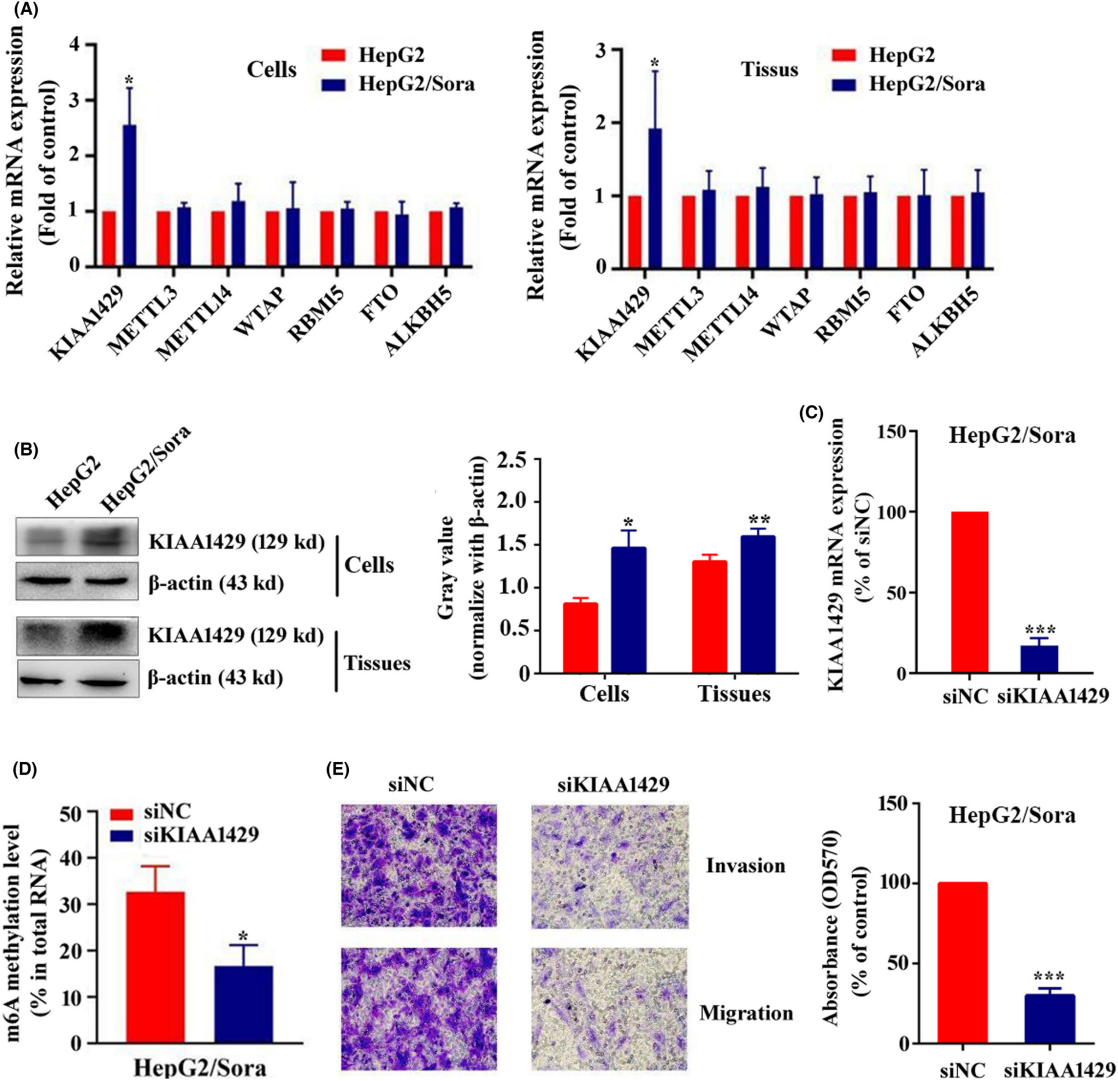
KIAA1429 Mediated the invasion, migration and EMT of sorafenib‐resistant strains. (A) RT‐qPCR assay. Relative mRNA expression levels of KIAA1429, METTL3, METTL4, WTAP, RBM15, FTO and ALKBH5 in HepG2 and HepG2/Sora cells or tissues. (B) Western blot analysis of KIAA1429 and β‐Actin in HepG2 and HepG2/Sora cells or tissues (left). Relative gray value of KIAA1429 according to western blot (right). HepG2 groups vs. HepG2/Sora groups, *p < 0.05, **p < 0.01. (C) RT‐qPCR assay. Relative mRNA expression of KIAA1429 in HepG2/Sora cells transfected with siNC or siKIAA1429. siNC groups vs. siKIAA1429 groups, ***p < 0.001. (D) m6A content in total RNA in HepG2/Sora cells transfected with siNC or siKIAA1429. siNC groups vs. siKIAA1429 groups, ***p < 0.001. (E) Transwell assay. Representative images (left, 100× magnification) and quantification (right) of HepG2/Sora cells migration after transfected with siNC or siKIAA1429. siNC groups groups vs. siKIAA1429 groups, ***p < 0.001.

Due to the upregulation of m6A methylation in sorafenib‐resistant strains (HepG2/Sora), the expression differences in m6A methylation‐related enzymes (METTL3, METTL14, WTAP, RBM15, KIAA1429, FTO and ALKBH5) in HepG2 and HepG2/Sora cells were detected by RT‐qPCR. As shown in Figure 4A, compared with HepG2 cells and their transplanted tumors, only KIAA1429 mRNA expression was significantly increased in HepG2/Sora cells and their transplanted tumors, which were 2.56 ± 0.66 (*p* < 0.05) times and 1.92 ± 0.78 (*p* < 0.05) times higher than that in the control group. Western blot results also confirmed that KIAA1429 was upregulated in HepG2/Sora cells and tissues (Figure 4B). Silencing KIAA1429 in HepG2/Sora cells resulted in a significant decrease in KIAA1429 expression (Figure 4C). After silencing KIAA1429 in HepG2/Sora cells, the overall m6A methylation level was decreased significantly to 52.72 ± 18.31% (*p* < 0.05) of the control group (Figure 4D). Transwell assays showed that the invasion and migration abilities of HepG2/Sora cells were decreased after silencing KIAA1429 (Figure 4E).

### 3.5 Effect of KIAA1429 on the angiogenesis of sorafenib‐resistant strains

**FIGURE 5:**
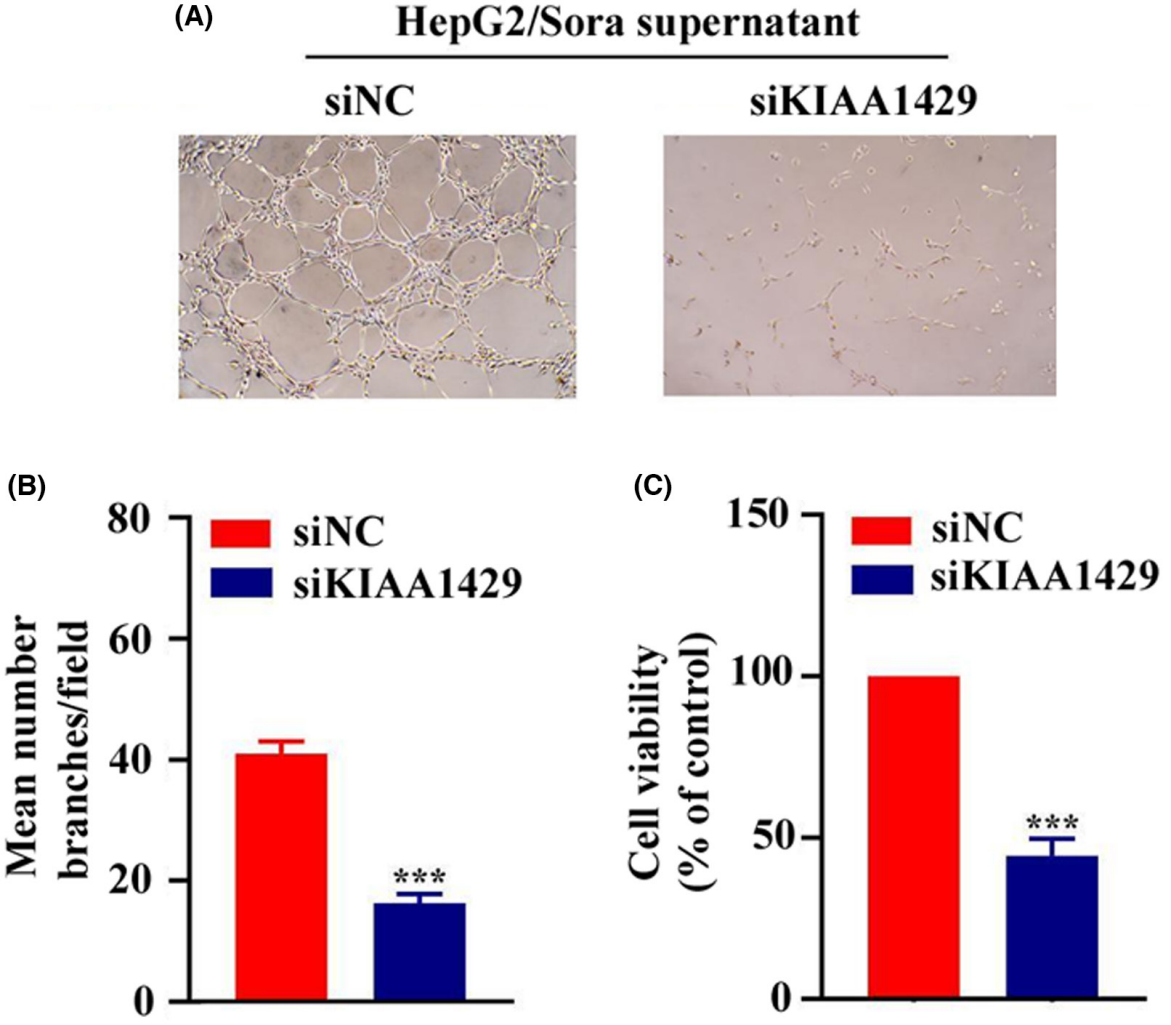
Effect of KIAA1429 on the angiogenesis of sorafenib‐resistant strains. (A) The angiogenesis of EA.HY926 cells in culture supernatants of HepG2/Sora cells transfected with siNC or siKIAA1429 (200× magnification). (B) Image J software was used to calculate the number of angiogenic branches in different conditioned media. siNC groups vs. siKIAA1429 groups, ***p < 0.001. (C) CCK‐8 assay. Cell viability of EA. hy926 cells in the culture supernatants of HepG2/Sora cells transfected with siNC or siKIAA1429. siNC groups vs. siKIAA1429 groups, ***p < 0.001.

The culture supernatants of HepG2/Sora or HepG2/Sora silenced KIAA1429 cells were used to induce vascular formation of EA.hy926 cells on Matrigel. As shown in Figure 5A, the supernatant of HepG2/Sora cells was able to effectively induce EA.hy926 cells to form blood vessels, while the supernatant of HepG2/Sora cells silenced KIAA1429, resulting in the sparse formation of blood vessels under the same conditions. The number of tubules formed in EA.hy926 cells induced by the culture supernatants of HepG2/Sora cells with silenced KIAA1429 was 36.59 ± 4.62% (*p* < 0.001) of the control group, and the viability of EA.hy926 cells was 44.45 ± 4.32% (*p* < 0.001) of the control group.

## 4 DISCUSSION

Hepatocellular carcinoma (HCC) is extremely malignant and easy to infiltrate and metastasize.^17^ Sorafenib, as a multitarget kinase inhibitor, is a targeted therapeutic drug for HCC approved by the US FDA.^18^ Although sorafenib has a very obvious therapeutic effect on patients with advanced hepatocellular carcinoma, a certain degree of drug resistance will eventually appear. Some studies have shown that cells can develop antiapoptotic effects after EMT, which leading to drug tolerance.^19^ Long‐term treatment with chemotherapy drugs can also induce EMT in tumor cells.^20^ Ritu Shrestha et al.^21^ reported that sorafenib resistance in hepatocellular carcinoma is closely related to EMT and the expression of immune checkpoint. Inhibiting the expression of EMT and immune checkpoint can restore the sensitivity of hepatocellular carcinoma to sorafenib. Peng‐Fei Zhang et al.^22^ reported that overexpression of SNHG3 can induce EMT and sorafenib resistance in HCC cells. This study further found that SNHG3 overexpression induced EMT in hepatoma cells through miR‐128/CD151 cascade activation. In our study, we confirmed that sorafenib‐resistant HepG2 cells showed a high degree of invasion and migration, accompanied by EMT phenomenon, which is consistent with some previous reports. We further found that EMT in sorafenib resistance was closely related to m6A methylation.

N6 methyladenosine (m6A) methylation occurs in approximately 25% of transcripts and is a common RNA methylation modification.^23^ It regulates RNA localization, transport, shearing, translation and maintenance of RNA stability at the transcriptional level.^14^, ^24^, ^25^ KIAA1429, as a member of the KIAA family, is the core protein of m6A methyltransferase and participates in the modification of m6A methylation.^26^ Several studies have investigated the role of KIAA1429‐mediated m6A methylation in the occurrence and development of hepatocellular carcinoma. Using bioinformatics, Xiaomin Wu et al.^27^ and Zedong Li et al.^28^ both reported that KIAA1429 is upregulated in HCC patients, and is closely related to the prognosis of patients using bioinformatics. Tian Lan et al.^29^ further found that KIAA1429 regulates the proliferation and migration of HCC through m6A methylation of GTAT3. In addition, KIAA1429 and its mediated m6A methylation play a promoting role in several cancers, such as colorectal cancer,^30^ breast cancer,^31^ etc. Compared with previous studies, we further revealed the role of KIAA1429 and its mediated m6A methylation in sorafenib resistant HCC. In our study, we further found that the EMT phenomenon of sorafenib‐resistant strains was regulated by m6A methylation, and the enzyme mediating m6A methylation was KIAA1429. Silencing KIAA1429 or inhibiting m6A methylation can inhibit the invasion, migration and EMT of sorafenib‐resistant hepatocellular carcinoma. We further found that silencing KIAA1429 could cause a decrease in angiogenesis in sorafenib‐resistant hepatocellular carcinoma. We suggest that the invasion and migration abilities of HCC are enhanced after sorafenib resistance, accompanied by EMT phenomenon, which may be related to the susceptibility of HCC patients with sorafenib resistance to cancer metastasis. The abnormal upregulation of KIAA1429 and its mediated m6A methylation modification in sorafenib‐ resistant hepatocellular carcinoma may be a key factor leading to its EMT.

Our experiment still has some shortcomings. Simple cell experiments have difficulty simulating the complex pathogenesis of hypertrophic cardiomyopathy. The AngII‐induced myocardial hypertrophy model of AC16 cells can only simulate a pathological phenomenon of hypertrophic cardiomyopathy, which does not represent hypertrophic cardiomyopathy. Therefore, we will introduce an animal model of hypertrophic cardiomyopathy in subsequent experiments to better study the pathogenesis of hypertrophic cardiomyopathy. In summary, our study revealed the molecular mechanism by which KAA1429 promotes EMT in sorafenib‐resistant HCC by mediating m6A methylation. KIAA1429, as a potential pathogenic molecule, may lead to tumor metastasis in patients with sorafenib‐resistant hepatocellular carcinoma. KIAA1429 is expected to become a new therapeutic and detection target.

## AUTHOR CONTRIBUTIONS

**Ye Kuang:** Writing – original draft (equal); writing – review and editing (equal). **Yun Cheng:** Data curation (equal); investigation (equal). **Jia Wang:** Formal analysis (equal); software (equal). **Hongyan Li:** Formal analysis (equal); resources (equal). **Xianghong Cao:** Formal analysis (equal); resources (equal). **Yang Wang:** Writing – original draft (lead); writing – review and editing (lead).

## ETHICAL APPROVAL

This study was approved by The Ethics Committee of Yan'an hospital of Kunming city (approval no. 2021‐03‐01).

## CLINICAL TRIAL REGISTRATION NUMBER

Not applicable.

## Data Availability

Data available on request from the authors.
